# Associations between ischaemic stroke subtypes and physical functioning in the first year post-stroke: a patient-reported outcome study

**DOI:** 10.1186/s41687-026-01063-8

**Published:** 2026-04-11

**Authors:** Tim Hagleitner, Dennis Freuer, Michael Ertl, Markus Naumann, Jakob Linseisen, Christa Meisinger

**Affiliations:** 1 Epidemiology, Medical Faculty, University of Augsburg, 86156 Augsburg, Germany; 2 Institute for Medical Information Processing, Biometry, and Epidemiology – IBE, LMU Munich, 81377 Munich, Germany; 3 Department of Neurolgy and Neurological Rehabilitation, District Hospital Guenzburg, 89312 Guenzburg, Germany; 4 Department of Neurology and Clinical Neurophysiology, University Hospital Augsburg: Universitatsklinikum Augsburg, 86156 Augsburg, Germany

**Keywords:** Ischaemic stroke, Etiology, Patient reported outcome measures, Quality of life, Activities of daily living, Motor activity

## Abstract

**Background:**

Ischaemic stroke etiology, as defined by the Trial of Org 10172 in Acute Stroke Treatment (TOAST) classification, has been associated with differences in survival and functional outcomes when assessed using the modified Rankin Scale (mRS). This study investigated whether similar patterns are reflected in patient-reported physical functioning during the first year post-stroke.

**Methods:**

We analysed data from the Stroke Cohort Augsburg (SCHANA), a prospective study of adults with confirmed ischaemic stroke treated at a tertiary care hospital in Southern Germany between 2018 and 2022. Physical functioning was assessed at 3- and 12-month post-discharge by using the physical domain score of the Stroke Impact Scale (SIS). Associations with stroke etiology were examined using linear mixed-effects models to improve precision and account for variable follow-up availability.

**Results:**

The final analytic sample included 1,044 patients and was characterised by predominantly mild neurological impairment at admission (median NIHSS 2.0). In adjusted linear mixed-effects models, stroke etiology was not significantly associated with patient-reported physical functioning. No etiologic subgroup differed significantly from the cardioembolic reference group (all *p* > 0.05).

**Conclusions:**

Among patients with predominantly mild ischaemic stroke, patient-reported physical functioning varied little across TOAST-defined subtypes. This may reflect limited between-group variation in stroke severity at baseline and conceptual differences between patient-reported and clinician-assessed outcome measures such as the mRS.

**Supplementary Information:**

The online version contains supplementary material available at 10.1186/s41687-026-01063-8.

## Background

Ischaemic stroke continues to represent a major global health burden, accounting for approximately 3.6 million deaths and 70 million disability-adjusted life years (DALYs) annually. In the post-pandemic era, it ranks third in global mortality and eighth in DALYs among level four causes, which denote the most specific diagnostic categories in the Global Burden of Disease study [1, 2].

Compared to 1990, age-standardised epidemiologic burden indicators have declined across most world regions [3], with further improvements projected for the years ahead [4]. Despite these favourable trends, the absolute number of individuals affected by ischaemic stroke is expected to rise markedly in the coming decades [4, 5]. This increase is driven by population ageing and the growing number of individuals living with the long-term consequences of stroke, reflecting continued improvements in acute stroke care [6–8]. As the population of individuals with a history of ischaemic stroke grows, interest in the factors that shape long-term outcomes is increasing. Stroke etiology may represent such a relevant factor in this context.

Ischaemic strokes are most commonly classified by underlying cause using the Trial of Org 10172 in Acute Stroke Treatment (TOAST) criteria [9–11]. This classification is clinically relevant, as it informs secondary prevention strategies [12, 13] and provides prognostic insights. Indeed, studies have shown that stroke subtypes predict long- and short-term survival and functional outcomes. This is illustrated by the fact that strokes caused by cardioembolism (CE) are typically more severe and carry higher mortality risk than other subtypes, both acutely and over the long term [14, 15]. Conversely, strokes resulting from small-vessel occlusion (SVO) tend to present with milder deficits and are generally linked to more favorable prognoses [14, 16].

Notably, the existing literature on stroke etiology and functional outcome has almost exclusively employed clinician-assessed instruments, with the modified Rankin Scale (mRS) being the most commonly used [17]. While the mRS, ranging from 0 (no symptoms) to 6 (death), remains a cornerstone measure of global disability in stroke research, it has well-recognized limitations. First, its broad categorical endpoints are relatively coarse, often requiring additional instruments to more precisely capture the degree and nature of residual functional impairment [18]. Second, structured and unstructured assessments of the mRS have been shown to yield inconsistent ratings at the extremes of the outcome spectrum [19].

Consequently, patient-reported outcome measures (PROMs) may offer valuable complementary insights by capturing dimensions of post-stroke functioning not accessible through clinician-administered instruments. They provide a patient-centered account of recovery that reflects lived experience and perceived limitations, and they may be more sensitive to subtle residual deficits in individuals [18]. Building on this rationale, the present study examines whether ischemic stroke etiology, classified according to the TOAST criteria, is associated with patient-reported physical functioning as measured by the Stroke Impact Scale (SIS) [20].

## Methods

### Study design and study population

This analysis is based on data from the Stroke Cohort Augsburg (SCHANA), a prospective, single-center cohort study conducted by the Chair of Epidemiology and the Department of Neurology and Clinical Neurophysiology at the University Hospital of Augsburg. As a regional center for specialized stroke care, the hospital admits approximately 2,000 stroke patients annually from the city of Augsburg and its surrounding areas.

The study population comprises all patients admitted to the hospital with a confirmed diagnosis of ischaemic or haemorrhagic stroke during two recruitment periods: SCHANA (September 2018 to November 2019) and SCHANA2 (January 2020 to May 2022). Adults aged 18 years or older were eligible for inclusion, regardless of whether the event was a first-ever or recurrent stroke. The study was approved by the Ethics Committee of Ludwig-Maximilians-Universität Munich (reference number: 18–196) and conducted in accordance with the principles outlined in the Declaration of Helsinki [21]. Written informed consent was obtained from all participants. Patients unable or unwilling to provide consent were excluded unless a legal representative consented on their behalf.

### Data collection and follow-up

**Fig. 1:**
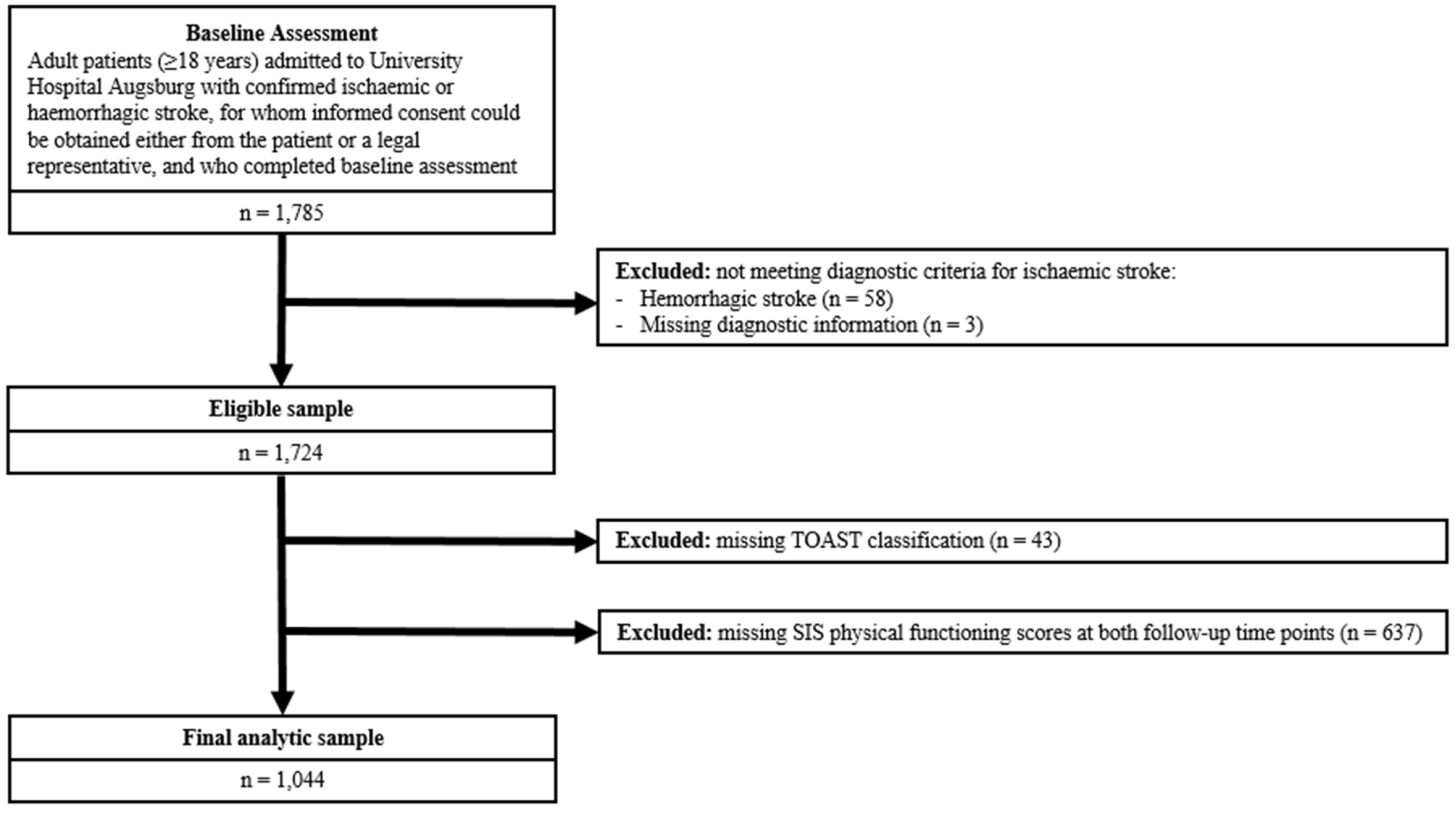
Flowchart of sample selection from the SCHANA cohort. Of 1,785 adult patients admitted to University Hospital Augsburg with confirmed ischaemic or hemorrhagic stroke, 61 were excluded due to non-ischaemic stroke or missing diagnostic information. After excluding further patients with missing TOAST classification (*n* = 43) and missing SIS physical functioning scores at both follow-ups (*n* = 637), the final analytic sample comprised 1,044 participants

Baseline data were collected during the acute hospital stay through structured interviews and were supplemented by systematic reviews of medical records. These covered sociodemographic characteristics, diagnostic findings, laboratory parameters, treatment modalities, and comorbidities. Follow-up data were obtained via standardized postal questionnaires administered at 3- and 12-months after hospital discharge. These surveys assessed lifestyle factors, functional status, and patient-reported outcomes. Detailed information on recruitment, data collection procedures, and follow-up measures is available in the published study protocol [21]. For the present analysis, only patients with a confirmed diagnosis of ischaemic stroke were included. A total of 1,724 patients met the general inclusion criteria and comprised the initial study sample (see Fig. 1). After exclusion of patients with missing information during follow-up at 3 and 12 months, 1,044 patients were finally included in this evaluation.

### Measurement of study variables

Physical quality of life was assessed using the SIS 2.0, a multidimensional, stroke-specific self-report measure. It covers eight domains of stroke-related disability and health-related quality of life, including physical, cognitive, and psychosocial functioning [22]. Although the SIS was later refined into version 3.0 to enhance measurement precision, the structure and content of the core physical domains remained unchanged [23]. For the present study, the validated German version was used [20]. SIS 2.0 and its successor have demonstrated good reliability, including strong internal consistency [24, 25] and test-retest reliability [26, 27], as well as solid construct [25] and concurrent validity [25, 27].

The primary outcome was post-stroke physical functioning, operationalized as the composite score of the physical domain of the SIS. This score combines four conceptually related subdomains: strength, hand function, mobility, and activities of daily living, into a single indicator of overall physical functioning. All subdomains and the composite score range from 0 to 100, with higher values reflecting better function and fewer perceived limitations [22]. Descriptive analyses were conducted for both the subdomains and the composite score to characterize functional outcomes and explore potential differences across specific aspects of physical functioning. Inferential analyses focused only on the composite score, as it provides a psychometrically robust and conceptually unified measure of physical functioning and helps reduce the risk of type I error. Previous research suggests that analyzing individual subdomains separately yields limited additional clinical value [27] and that the composite score offers superior discriminatory capacity compared to its constituent subdomains [23].

Stroke etiology was classified according to the TOAST criteria, based on an integration of findings from neuroimaging, vascular and cardiac diagnostics, laboratory testing, and clinical assessment. This mechanism-based classification distinguishes five subtypes of ischaemic stroke: cardioembolism (CE), large-artery atherosclerosis (LAA), small-vessel occlusion (SVO), other determined causes (OC), and cryptogenic causes (CC) [11].

Baseline sociodemographic variables included age, sex, employment status, and educational attainment, with the latter classified according to the International Standard Classification of Education (ISCED-97) [28]. Based on this classification, educational level was dichotomised into low (ISCED levels 0 to 3) and high (ISCED levels 4 to 6). Employment status was categorised as employed or not employed.

Lifestyle-related factors assessed at baseline comprised smoking behaviour, alcohol consumption, physical activity, and BMI. Smoking status was categorized as never, former, or current smoker. Alcohol use was evaluated using the Alcohol Use Disorders Identification Test (AUDIT) [29]. Physical activity was assessed with the categorical score of the short form of the International Physical Activity Questionnaire (IPAQ) [30] according to the standardized scoring method [31].

Multimorbidity was defined using the Multimorbidity-Weighted Index (MWI), a validated, person-centered measure that captures disease burden based on the cumulative functional impact of chronic conditions [32]. The measure was selected because it reflects the specific impact of chronic disease burden on physical functioning, making it more appropriate than mortality-oriented comorbidity indices [33]. In the present study, chronic conditions included in the index and assessed at baseline were mapped using the recently published ICD-10–based weights of the MWI [34] (see Supplementary Table S1).

### Statistical methods

Baseline characteristics were descriptively summarized for patients stratified by stroke etiology. Continuous variables were reported as medians with interquartile ranges (IQR), and categorical variables as absolute and relative frequencies. Group differences were examined using the Pearson’s chi-square test for categorical variables and the Kruskal–Wallis test for ordinal and continuous variables. Where applicable, significant results were followed by post hoc pairwise comparisons using Dunn’s test with Bonferroni correction.

Differences in SIS physical domain and subdomain scores across etiologic groups were visualized using boxplots. For the physical domain, group differences of SIS scores were analyzed using Kruskal–Wallis tests, followed by Dunn’s post hoc tests with Bonferroni adjustment.

To examine associations between stroke etiology and physical domain scores in this longitudinal study with up to three measurements per participant, a linear mixed-effects model with a random intercept for each individual was fitted. Participant characteristics were assessed at baseline, and outcome data were available from the 3- and 12-month follow-ups (at least one follow-up). This modelling approach accounted for varying follow-up availability, thereby increasing the effective sample size and enhancing precision. CE subtype was chosen as the reference category due to its relatively large case number and greater etiologic homogeneity compared to CC. Besides etiology, covariates identified as confounders were included as fixed effects. Covariate selection was guided by a directed acyclic graph (DAG) illustrating assumed causal relationships between etiology and physical functioning (see Supplementary Figure S1 and Supplementary Table S2). The DAG was constructed using the Dagitty web application (version 3.0) [35] based on theoretical assumptions derived from a literature review. Notably, it posits both a potential direct effect of stroke etiology on physical outcomes and an indirect effect mediated by stroke severity.

Interaction terms between etiology groups and sex were included in exploratory models to assess potential effect modification. However, none reached statistical significance, and they were therefore not retained in the final models. All continuous variables were z-standardized before inclusion and before generating polynomial terms. To improve model fit and account for potential non-linear relationships, higher-order polynomial terms were subsequently added. Inclusion of these terms was guided by likelihood ratio tests comparing nested models with and without the respective terms. Assumptions of normality, homoscedasticity, linearity, and multicollinearity were assessed using standard diagnostic tools, including Q–Q plots of residuals and random effects, residual-vs-fitted plots, and variance inflation factors. All analyses were conducted in R (version 4.4.2) using a two-sided significance level of 0.05.

## Results

Of the initial 1,724 cases, 680 (39.4%) were excluded due to missing etiological classification or absence of SIS physical domain scores at both follow-up time points. The final analytical sample comprised 1,044 cases. Compared to excluded individuals, participants included in the analytical sample were significantly younger and more frequently male. No significant differences were observed with regard to stroke etiology or multimorbidity (see Supplementary Table S3).

### Baseline characteristics

The final analytical sample was characterized by a median age of 71.0 years (IQR 60.0–78.0), with men representing the majority of participants (59.3%). The most common stroke etiology was CC (34.7%), followed by CE (24.1%), LAA (21.6%), and SVO (17.6%). Due to limited case numbers and etiological heterogeneity, the OC subtype (1.9%) was excluded from all descriptive group comparisons. The median stroke severity at admission, measured using the National Institutes of Health Stroke Scale (NIHSS) [36], was 2.0 (see Table 1).

Multimorbidity was highest in the CE group and lowest in the CC group. Post hoc comparisons revealed a higher multimorbidity burden in patients with CE compared to those with CC (*p* < 0.001), LAA (*p* < 0.001), and SVO (*p* = 0.028). Conversely, patients with CC had lower multimorbidity than those with SVO (*p* = 0.028). Stroke etiology also differed by sex (*p* = 0.006). Based on within-sex distribution CE and CC strokes were more common in women (26.6% and 38.1%, respectively) than in men (22.5% and 32.3%, respectively), whereas LAA and SVO strokes were more prevalent in men. No other covariates differed notably between etiologic subgroups (see Table 2).

**Table 1:** Stroke severity (NIHSS) and functional outcome (mRS) stratified by etiology subgroups

NIHSS^2^	Etiology^1^											*p*
	Total	CE		LAA		SVO		CC		OC		
Admission, Median (*IQR*^†^) *n* = 994	2.0 (*0.0–4.0*)	2.0 (*1.0–6.0*)		2.0 (*0.0–4.0*)		2.0 (*0.0–4.0*)		1.0 (*0.0–3.0*)		1.0 (*0.0–2.0*)		< 0.001^a^
*Significant pairwise differences* ^*b*^	*CE – LAA (p = 0.018)*,* CE – CC (p < 0.001)*,* SVO – CC (p = 0.012)*											
Discharge, Median (*IQR*) *n* = 942	0.0 (*0.0–2.0*)	0.0 (*0.0–1.0*)		1.0 (*0.0–2.0*)		1.0 (*0.0–2.0*)		0.0 (*0.0–1.0*)		0.0 (*0.0–1.0*)		< 0.001^a^
*Significant pairwise differences* ^*b*^	*LAA – CC (p = 0.014)*,* SVO – CC (p < 0.001)*											

**mRS** ^3^	**Etiology**										**p**	
	**Total**		**CE**	**LAA**	**SVO**		**CC**		**OC**			
Admission, Median (*IQR*) *n* = 991	2.0 (*1.0–3.0*)		3.0 (*1.0–4.0*)	2.0 (*1.0–3.0*)	2.0 (*0.0–4.0*)		2.0 (*1.0–3.0*)		2.0 (*1.0–2.0*)		< 0.001^a^	
*Significant pairwise differences* ^*b*^	*CE – LAA (p = 0.028)*,* CE – SVO (p = 0.024)*,* CE – CC (p < 0.001)*,* LAA – CC (p = 0.045)*											
Discharge, Median (*IQR*) *n* = 987	1.0 (*0.0–2.0*)		1.0 (*0.0–2.0*)	1.0 (*0.0–2.0*)	1.0 (*0.5–2.0*)		1.0 (*0.0–2.0*)		0.5 (*0.0–1.0*)		< 0.001^a^	
*Significant pairwise differences* ^*b*^	*CE – CC (p = 0.011)*,* LAA – CC (p = 0.014)*,* SVO – CC (p < 0.001)*											

Note

^1^ Etiology was classified according to the TOAST criteria: CE = cardioembolism; LAA = large-artery atherosclerosis; SVO = small-vessel occlusion; CC = cryptogenic cause; OC = other cause. ^2^ National Institutes of Health Stroke Scale (NIHSS), range 0–42; higher scores indicate greater neurological deficit. ^3^ Modified Rankin Scale (mRS), range 0–6; higher scores indicate greater disability, with 6 representing death. ^a^ Kruskal–Wallis Tests excluding ‘OC’ category. ^b^ Dunn test with Bonferroni adjusted p values excluding ‘OC’ category. ^†^ Interquartile range

**Table 2:** Baseline sample characteristics stratified by etiology subgroups

Variable		Etiology^1^							*p*
		Total	CE	LAA	SVO	CC	OC		
Etiology, n (*row%*) *n* = 1.044			252 (*24.1%*)	226 (*21.6%*)	184 (*17.6%*)	362 (*34.7%*)	20 (*1.9%*)		
Age, Median (*IQR*^†^) *n* = 1.044		71.0 (*60.0–78.0*)	74.0 (*63.0–80.0*)	70.0 (*62.0–78.0*)	68.0 (*59.0–78.0*)	70.5 (*60.0–78.0*)	48.0 (*40.5–58.0*)	0.051^a^	
Alcohol,^2^ Median (*IQR*) *n* = 989		2.0 (*1.0–4.0*)	2.0 (*1.0–4.0*)	3.0 (*1.0–4.0*)	2.0 (*1.0–4.0*)	2.0 (*1.0–4.0*)	2.0 (*1.0–4.0*)	0.554^a^	
BMI, Median (*IQR*) *n* = 973		26.5 (*24.2–30.0*)	26.2 (*24.2–30.0*)	26.2 (*24.2–29.4*)	27.2 (*24.5–30.9*)	26.4 (*23.9–30.1*)	27.0 (*23.9–30.1*)	0.203^a^	
Education,^3^ n (*col%*) *n* = 992	Low	740 (*74.6%*)	181 (*75.4%*)	166 (*78.3%*)	134 (*76.6%*)	246 (*70.7%*)	13 (*76.5%*)	0.189^b^	
	High	252 (*25.4%*)	59 (*24.6%*)	46 (*21.7%*)	41 (*23.4%*)	102 (*29.3%*)	4 (*23.5%*)		
Employment status, n (*col%*) *n* = 1.025	Employed	289 (*28.2%*)	61 (*24.3%*)	56 (*25.5%*)	51 (*28.3%*)	107 (*30.0%*)	14 (*82.4%*)	0.408^b^	
	Unemployed	736 (*71.8%*)	190 (*75.7%*)	164 (*74.5%*)	129 (*71.7%*)	250 (*70.0%*)	3 (*17.6%*)		
Multimorbidity,^4^ Median (*IQR*) *n* = 1.044		2.87 (*1.9–5.0*)	3.78 (*2.7–6.1*)	2.77 (*1.9–4.5*)	2.69 (*1.9–4.9*)	1.87 (*1.5–4.4*)	1.07 (*0.6–2.8*)	< 0.001^a^	
Physical activity,^5^ n (*col%*) *n* = 1.044	Low	516 (*49.5%*)	126 (*50.0%*)	111 (*49.1%*)	104 (*56.5%*)	167 (*46.1%*)	8 (*40.0%*)	0.222^b^	
	Moderate	278 (*26.6%*)	59 (*23.4%*)	62 (*27.4%*)	47 (*25.5%*)	105 (*29.0%*)	5 (*25.0%*)		
	High	250 (*23.9%*)	67 (*26.6%*)	53 (*23.5%*)	33 (*17.9%*)	90 (*24.9%*)	7 (*35.0%*)		
Sex, n (*col%*) *n* = 1.044	Male	619 (*59.3%*)	139 (*55.2%*)	152 (*67.3%*)	119 (*64.7%*)	200 (*55.2%*)	9 (*45.0%*)	0.006^b^	
	Female	425 (*40.7%*)	113 (*44.8%*)	74 (*32.7%*)	65 (*35.3%*)	162 (*44.8%*)	11 (*55.0%*)		
Smoking, n (*col%*) *n* = 1.044	Never	463 (*44.3%*)	116 (*46.0%*)	87 (*38.5%*)	82 (*44.6%*)	167 (*46.1%*)	11 (*55.0%*)	0.519^b^	
	Former	456 (*43.7%*)	111 (*44.0%*)	106 (*46.9%*)	80 (*43.5%*)	151 (*41.7%*)	8 (*40.0%*)		
	Current	125 (*12.0%*)	25 (*9.9%*)	33 (*14.6%*)	22 (*12.0%*)	44 (*12.2%*)	1 (*5.0%*)		

Note:^1^ Etiology was classified according to the TOAST criteria: CE = cardioembolism; LAA = large-artery atherosclerosis; SVO = small-vessel occlusionCC = cryptogenic cause; OC = other cause (excluded in all tests)^2^ Alcohol consumption was assessed using the Alcohol Use Disorders Identification Test^3^ Education was assessed according to the ISCED-97 classification and dichotomized into low (ISCED levels 0–3) and high (ISCED levels 4–6)^4^ Multimorbidity was assessed using the summed beta coefficients of the Multimorbidity Weighted Index (MWI).^5^ Physical activity was assessed using the categorical score of the short-form International Physical Activity Questionnaire^a^ Kruskal–Wallis Test^b^ Pearson’s chi-squared test^†^ Interquartile range

### Stroke impact scale domain scores

Across all domains (physical domain and subdomains), the score distributions were generally non-modal, except for the strength. Score distributions at 3- and 12-month follow-up were highly similar within each domain (see Supplementary Figure S2).

**Fig. 2:**
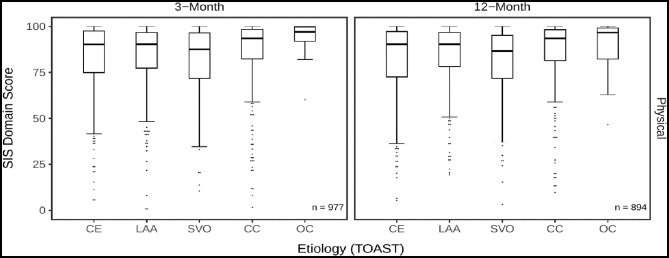
Boxplots of SIS physical domain scores stratified by etiology subgroups. Scores are shown at 3- and 12-month. Boxplots depict the median, interquartile range (IQR), and whiskers extending to the most extreme values within 1.5 times the IQR. Outliers are shown as filled dots; vertical stacking indicates repeated values at the same score. SIS domain scores are stratified by ischemic stroke etiology according to the TOAST classification: CE = cardioembolism, LAA = large-artery atherosclerosis, SVO = small-vessel occlusion, CC = cryptogenic cause, OC = other determined causes

In the physical domain, 13.1% of the patients reached the maximum score at 3 months, and 13.8% did so at 12 months. Median scores at both time points were highest in the CC and OC groups, with elevated values also observed at the 25th percentile. The 75th percentile remained just below the maximum score across all groups, with only minor differences between them. The SVO group showed comparatively lower scores at both time points, while the CE and LAA groups fell within an intermediate range (see Fig. 2; Table 3). Unadjusted associations between etiology groups and physical domain scores were statistically significant at both 3- and 12-month follow-up (*p* < 0.001). Post hoc comparisons at 3 months indicated differences between CC and LAA (*p* = 0.037) and between CC and SVO (*p* < 0.001). At 12 months, notable differences were observed between CC and CE (*p* = 0.040) and between CC and SVO (*p* < 0.001).

**Fig. 3:**
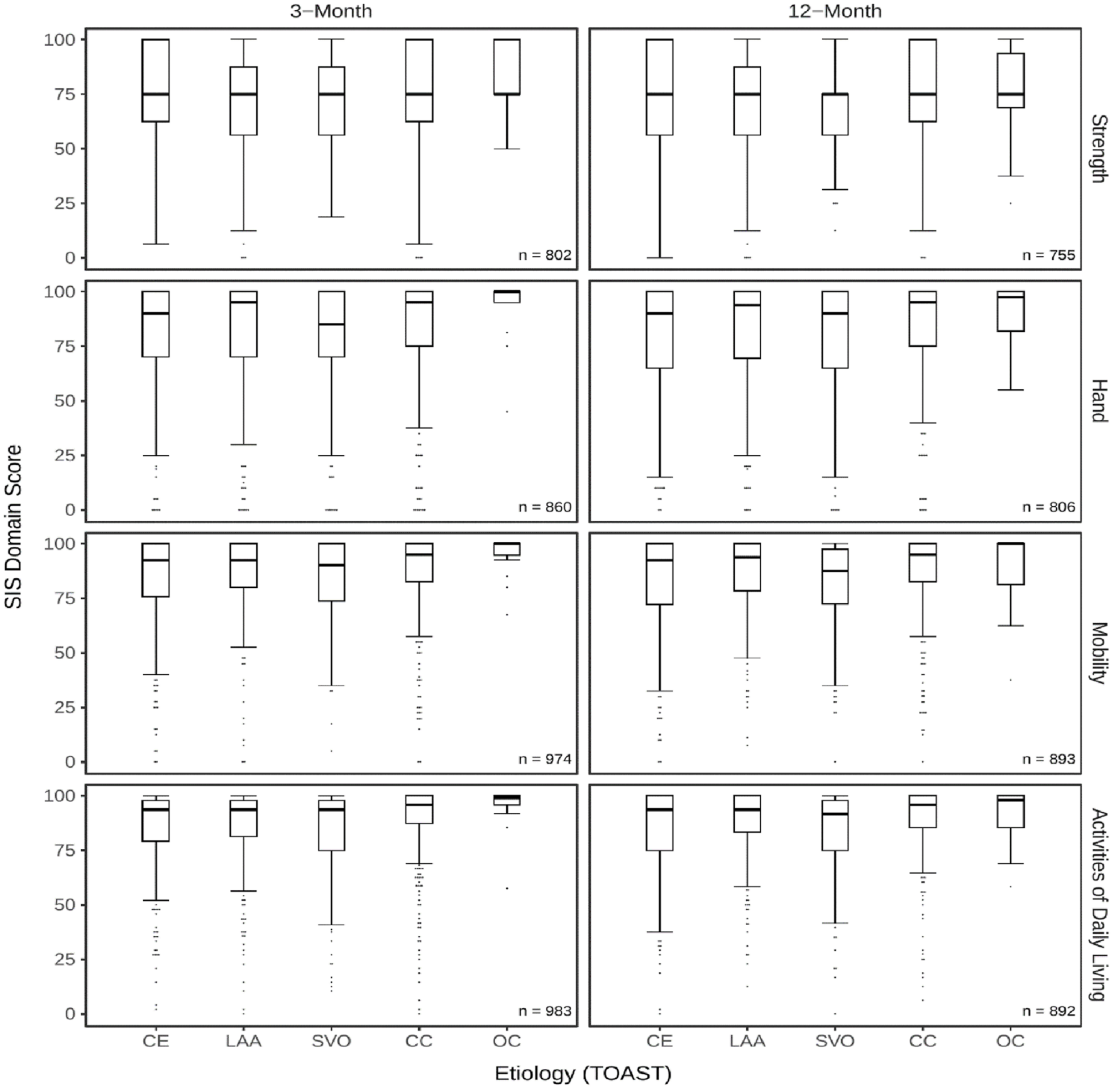
Boxplots of SIS subdomain scores stratified by etiology subgroups. Scores are shown at 3- and 12-month. Boxplots depict the median, interquartile range (IQR), and whiskers extending to the most extreme values within 1.5 times the IQR. Outliers are shown as filled dots; vertical stacking indicates repeated values at the same score. SIS domain scores are stratified by ischemic stroke etiology according to the TOAST classification: CE = cardioembolism, LAA = large-artery atherosclerosis, SVO = small-vessel occlusion, CC = cryptogenic cause, OC = other determined causes

In the subdomains, ceiling effects were more pronounced than in the physical domain, with 22.0% of patients reaching the maximum score in strength at 12 months and 42.9% in hand function at 3 months. Overall, subdomain score distributions mirrored those of the physical domain (see Fig. 3).

**Table 3:** Unadjusted associations between etiology subgroups and physical domain scores

Physical domain scores	Etiology^1^						*p*
	Total	CE	LAA	SVO	CC	OC	
3-month follow-up, Median (*IQR*^†^) *n* = 977	91.1 (*77.4–97.4*)	90.3 (*75.0–97.6*)	90.3 (*77.4–96.8*)	87.5 (*71.8–96.6*)	93.5 (*82.4–98.4*)	97.0 (*91.9–99.8*)	< 0.001^a^
*Significant pairwise differences* ^*b*^	*LAA – CC (p = 0.037)*,* SVO – CC (p < 0.001)*						
12-month follow-up, Median (*IQR*) *n* = 894	91.1 (*76.6–96.8*)	90.3 (*72.6–97.2*)	90.3 (*78.2–96.8*)	86.7 (*71.8–95.2*)	93.5 (*81.5–98.1*)	96.8 (*82.3–99.2*)	< 0.001^a^
*Significant pairwise differences* ^*b*^	*CE – CC (p = 0.040)*,* SVO – CC (p < 0.001)*						

Note:^1^ Etiology was classified according to the TOAST criteria: CE = cardioembolism; LAA = large-artery atherosclerosis; SVO = small-vessel occlusion; CC = cryptogenic cause; OC = other cause^a^ Kruskal–Wallis Tests excluding ‘OC’ category^b^ Dunn test with Bonferroni adjusted p values excluding ‘OC’ category^†^ Interquartile range

### Linear mixed-effects model results

A total of 1,665 observations from 918 patients were included in the model, comprising 868 observations at the 3-month follow-up and 797 at the 12-month follow-up. Fixed effects, including stroke etiology, sociodemographic, and lifestyle variables, explained 23% of the variance in physical domain scores, as indicated by the marginal R². The conditional R² was 0.90, and the intraclass correlation coefficient from the adjusted model was 0.87. Residual diagnostics indicated mild heteroscedasticity. To address this and ensure robust inference, cluster-robust standard errors (CR2 method, clubSandwich package) [37] were applied. Overall, the model conformed well to the assumptions of the linear mixed-effects framework.

In the adjusted linear mixed-effects model, no associations were found between etiology subtypes and SIS physical domain scores. Effect sizes compared to the CE group were small and non-significant (all *p* > 0.05; see Table 4). Standard errors for stroke etiology and covariates differed only marginally between models using cluster-robust and conventional estimation. Complete fixed-effects results are available in Supplementary Table S4.

**Table 4:** Fixed effects estimates for etiology subgroups: results from the linear mixed model

Variable		β^†^ (SE^‡^)	95% CI^§^	*p*
Etiology^1^	CE (*reference group*)			
	LAA	-0.46 (*1.83*)	[-4.05, 3.13]	0.801
	SVO	-1.92 (*1.79*)	[-5.44, 1.61]	0.286
	CC	1.98 (*1.50*)	[-0.97, 4.92]	0.187
	OC	0.41 (*3.49*)	[-6.89, 7.70]	0.909

Note:^1^ Etiology was classified according to the TOAST criteria: CE = cardioembolism; LAA = large-artery atherosclerosis;SVO = small-vessel occlusion; CC = cryptogenic cause; OC = other causes^†^ Beta Regression Coefficient^‡^ Standard Error (cluster-robust CR2 type)^§^ Confidence Interval

## Discussion

### Principal findings

No notable associations were observed between ischaemic stroke etiology, as defined by the TOAST classification, and patient-reported physical functioning. The estimated beta coefficients, representing differences in SIS physical domain scores, were all below 2.0 points relative to the CE reference group. Differences of this magnitude are unlikely to be clinically meaningful. For comparison, a previous study identified a clinically important difference of 4.5 points for the SIS 3.0 mobility subdomain, the lowest reported threshold among the physical domains [38].

### Interpretation in the context of previous evidence

Previous research suggests that ischaemic stroke subtypes, as classified by the TOAST criteria, are associated with different clinical outcomes. CE strokes have been linked to higher mortality rates than LAA and SVO, both in the short term [16, 39] and over longer follow-up periods extending several years [14, 15]. SVO strokes are associated with favourable survival rates [14, 15, 39] and long-term functional outcomes as assessed by the mRS [14]. This likely reflects their characteristically mild clinical presentation, as SVO strokes predominantly involve small penetrating arteries and typically spare larger cortical and subcortical regions [40].

One study reported better functional outcomes for patients with CC strokes relative to other subtypes, independent of baseline stroke severity [41].

Yet, studies relying on the mRS assess a different construct of physical outcome. This scale captures global disability and dependence in activities of daily living and is traditionally applied by clinicians or trained assessors using an unstructured approach [19]. It is weighted toward mobility, self-care, and overall independence, and relies on broad categorical endpoints [18]. In contrast, the SIS physical domain capture individuals’ perceived limitations and capabilities in everyday life, offering a more nuanced and person-centered assessment. Accordingly, the absence of an association in our findings does not necessarily contradict previous research but may rather reflect differences in outcome measures and methodology.

Severely affected individuals are less likely to be represented in patient-reported measures due to death, profound disability, or inability to self-report [18]. In contrast, the mRS more readily includes such cases via clinical or proxy assessment and captures the full range of outcomes, including death. Stroke severity at admission was low in our sample (median NIHSS 2.0, IQR 0.0 to 4.0), whereas higher values were reported in registry-based studies, such as 5.0 (2.0 to 10.0) in Lille, France [39], and 4.0 (2.0 to 8.0) in the federal state of Hesse, Germany [42]. Given the nature of these datasets, they likely reflect the general hospitalized stroke population more accurately.

Furthermore, the ordinal structure of the mRS, which is frequently dichotomized in analytical practice [17], may distort between-group differences. Small variations near category thresholds can appear exaggerated when individuals are assigned to adjacent outcome groups, while meaningful differences within a single broad category cannot be captured. In contrast, continuous scales such as the SIS are generally less prone to artificial threshold effects.

In addition, psychological processes such as adaptation and response shift, which contribute to the so-called disability paradox, may lead patients to rate their physical functioning more favourably over time, regardless of objective impairments [43]. In line with this, patients with good functional outcomes have been shown to self-report lower mRS scores on structured questionnaires when compared to those assigned by clinicians in unstructured evaluations [19].

### Strength and limitations

Key strengths of this study include its comparatively large analytical sample, which is notable for research on patient-reported physical functioning after ischaemic stroke. Second, the prospective cohort design, with assessed baseline characteristics and two defined follow-up points, enabled the use of linear mixed-effects models that incorporated repeated measures per participant. This approach allowed for flexible handling of varying follow-up availability. Finally, the analytical strategy was theory-driven, using a directed acyclic graph informed by existing literature to guide covariate selection and increase internal validity.

The findings of this study should be interpreted considering several potential limitations. First, practical challenges inherent to the use of patient-reported outcome measures are well known to introduce selection bias at both the initial recruitment stage and during follow-up assessments. The self-reporting of physical functioning relies on cognitive, communicative, and attentional abilities, which are often compromised in stroke survivors due to neuropsychological impairments, advanced age, or comorbid conditions [18].

A methodological limitation arises from the use of complete case analysis, which assumes that data are missing completely at random (MCAR). In the present context, this assumption is unlikely to hold, as missingness was plausibly related to the outcome itself. Patients with greater levels of impairment were less likely to complete follow-up assessments, introducing systematic bias and potentially attenuating associations between stroke etiology and physical functioning. Statistical approaches to handle data missing not at random (NMAR) exist but rely on strong, often unverifiable assumptions regarding the missingness mechanism [44]. These methods are not established in stroke research with patient-reported outcomes [18] and were not within the scope of this study.

Finally, the statistical power of the functional outcome analyses may have been limited. Although the sample size was considerable, the absence of a formal power calculation restricts the interpretability of non-significant results. This limitation is particularly relevant given the presence of considerable ceiling effects, which may have reduced sensitivity to between-group differences. However, the degree to which maximum scores represent actual recovery, as opposed to imprecision in measurement, cannot be determined.

Regarding generalizability, the findings are limited to patients with mild stroke severity and cannot be extrapolated to those with severe initial functional impairments. Moreover, as the study was conducted at a single tertiary stroke center, generalizability to other clinical settings or health systems may be limited by differences in diagnostic practices and patient characteristics.

### Conclusion

The limited variation in SIS outcomes may reflect a uniformly perceived level of physical recovery among patients with mild stroke severity, regardless of etiology. Two factors may account for the discrepancy compared to mRS-based studies: differences in the constructs captured, and the inclusion of more severely affected patients, including deaths, in those analyses. The latter may suggest that stroke etiology primarily shapes initial stroke severity, whereas the subsequent recovery trajectory, may not be influenced by the etiologic subtype. However, as stroke severity was conceptualized as a mediator and not adjusted for in the model, this interpretation remains speculative. Further research is needed to examine the extent to which similar patterns are observed in other cohorts and when using alternative patient-reported outcome measures assessing physical functioning and related subdomains (e.g. PROMIS [45] or SS-QOL [46]).

## Supplementary Information

Below is the link to the electronic supplementary material.

Supplementary Material 1

## Data Availability

The datasets used and analysed during the current study are available from the corresponding author on reasonable request.
