# Supplementary material for: Associations between ischaemic stroke subtypes and physical functioning in the first year post-stroke: a patient-reported outcome study
Source: J Patient Rep Outcomes. 2026 Apr 11;10:61. doi: 10.1186/s41687-026-01063-8 (PMC13083565; doi:10.1186/s41687-026-01063-8)
Supplement: Supplementary file 1 — Supplementary Material 1 [file 41687_2026_1063_MOESM1_ESM.docx]

**Associations between ischaemic stroke subtypes and physical functioning in the first year post-stroke: a patient-reported outcome study**

# Supplementary Material

| **Supplementary Table S1.** Chronic conditions assessed at baseline mapped to MWI categories and weights | | | |
| --- | --- | --- | --- |
| **Condition** (Standard Data Collection) | **Diagnostic Condition** (Multimorbidity-Weighted Index) | **Beta Coefficient**  (Multimorbidity-Weighted Index) |  |
| **Category: Cardiovascular** | | |  |
| Atrial flutter / Atrial fibrillation | Arrhythmias | 1.330 |  |
| Cardiomyopathy | Congestive heart failure, Cardiomyopathy | 4.770 |  |
| Congestive heart failure | Congestive heart failure, Cardiomyopathy | 4.770 |  |
| Coronary artery disease and myocardial infarction | Myocardial infarction | 1.730 |  |
| Disease of the heart valves | Valvular heart disease | 0.416 |  |
| Hypertension | High blood pressure, hypertension | 1.530 |  |
| **Category: Endocrine** | | |  |
| Diabetes mellitus | Diabetes mellitus | 2.670 |  |
| Elevated cholesterol and fat levels | Elevated cholesterol, Hyperlipidemia | 0.343 |  |
| **Category: Musculoskeletal** | | |  |
| Osteoporosis | Osteoporosis | 0.997 |  |
| **Category: Nervous** | | |  |
| Epilepsy | Epilepsy | 0.841 |  |
| Migraine | Migraine headache | 0.614 |  |
| **Category: Oncologic** | | |  |
| Malignant tumour / Cancer^a^ | Other cancer, Neoplasms of unspecified nature | 1.760 |  |
| **Category: Psychatric** | | |  |
| Anxiety disorder / Panic attack | Anxiety | 1.290 |  |
| Depression | Depression and related psychiatric conditions | 1.290 |  |
| **Category: Pulmonary** | | |  |
| Chronic bronchitis / Chronic obstructive pulmonary disease | Chronic pulmonary diseases | 4.320 |  |
| **Note.**  ^a^ In the absence of more specific diagnostic information on cancer type, all cancer cases in this study were assigned the MWI weight for “Other cancer, Neoplasms of unspecified nature” (−1.76), as defined in the original index. This category was used in accordance with the recommendation to assign the best-fitting available weight based on diagnostic resolution. Specific MWI weights for oncologic conditions range from 0.0 (e.g., basal cell carcinoma, squamous cell carcinoma) to −6.25 (lung cancer), with intermediate weights for other common types: breast cancer (−0.886), prostate cancer (−0.402), colorectal cancer (−1.18), and blood cancers such as leukemia or lymphoma (−1.32). The weight of −1.76 thus represents a conservative estimate for cancer-related multimorbidity burden in the absence of subtype classification. | | |  |

| **Supplementary Table S2.** Literature review informing the creation of the directed acyclic graph | | | | | |
| --- | --- | --- | --- | --- | --- |
| **Paths** | | | **Clarification** | **References** |  |
| Etiology (TOAST) | → | Physical Outcome | Assumed direct path | - |  |
| Etiology (TOAST) | → | Stroke Severity | Part of assumed indirect causal path | - |  |
| Stroke Severity | → | Physical Outcome | Part of assumed indirect causal path | - |  |
| Age | → | Etiology (TOAST) |  | [1] [2] |  |
| Age | → | Physical Outcome |  | [3] [4] |  |
| Sex | → | Etiology (TOAST) |  | [5] [6] |  |
| Sex | → | Physical Outcome | Literature suggests no causal effect^1^ | [7] [8] |  |
| Socioeconomic status | → | Physical Outcome |  | [9] [10] |  |
| Socioeconomic status | → | Diseases  (Causal risk factors for subtypes) |  | [9] [10] |  |
| Lifestyle factors^2^ | → | Etiology (TOAST) |  | [11] [12] [13] [14] |  |
| Lifestyle factors | → | Physical Outcome |  | [15] [16] |  |
| Diseases  (Causal risk factors for stroke subtypes) | → | Etiology (TOAST) |  | [17] [18] [19] |  |
| Diseases  (Causal risk factors for stroke subtypes) | → | Multimorbidity |  | [20] |  |
| Multimorbidity | → | Physical Outcome |  | [21] |  |
| **Other assumed paths (depicted for structural completeness)** | | | | |  |
| Age | → | Diseases  (Causal risk factors for subtypes) |  | [22] |  |
| Age | → | Diseases  (Non-causal risk factors for stroke subtypes) |  | - |  |
| Age | → | Stroke severity |  | [23] |  |
| Age | → | Socioeconomic status |  | - |  |
| Age | → | Lifestyle factors |  | - |  |
| Sex | → | Diseases  (Causal risk factors for subtypes) |  | [24] [25] |  |
| Sex | → | Diseases  (Non-causal risk factors for stroke subtypes) |  | [26] |  |
| Sex | → | Stroke severity |  | [27] |  |
| Sex | → | Socioeconomic status |  | - |  |
| Sex | → | Lifestyle factors |  | - |  |
| Socioeconomic status | → | Diseases  (Non-causal risk factors for stroke subtypes) |  | - |  |
| Socioeconomic status | → | Lifestyle factors |  | - |  |
| Lifestyle factors | ↔ | Diseases  (Causal risk factors for stroke subtypes) |  | - |  |
| Lifestyle factors | ↔ | Diseases  (Non-causal risk factors for stroke subtypes) |  | - |  |
| Diseases  (Non-Causal risk factors for stroke subtypes) | → | Multimorbidity |  | - |  |
| **Note.**  ^1^ No causal effect observed after adjustment for covariates. Nevertheless, sex is retained as a covariate in the model due to its  potential role as a confounder and standard practice in stroke research.  ^2^ Lifestyle factors in the context of this study includes alcohol consumption, smoking behaviour, physical activity and body mass index. | | | | |  |
| **References**  1. Schöberl F, Ringleb PA, Wakili R, Poli S, Wollenweber FA, Kellert L. Juvenile Stroke. Dtsch Arztebl Int. 2017; 114:527–34.  2. Nacu A, Fromm A, Sand KM, Waje-Andreassen U, Thomassen L, Naess H. Age dependency of ischaemic stroke subtypes and vascular risk factors in western Norway: the Bergen Norwegian Stroke Cooperation Study. Acta Neurol Scand. 2016; 133:202–7.  3. Ohya Y, Matsuo R, Sato N, Irie F, Wakisaka Y, Ago T, et al. Modification of the effects of age on clinical outcomes through management of lifestyle-related factors in patients with acute ischemic stroke. J Neurol Sci. 2023; 446:120589.  4. Yoo JW, Hong BY, Jo L, Kim J-S, Park JG, Shin BK, et al. Effects of Age on Long-Term Functional Recovery in Patients with Stroke. Medicina (Kaunas). 2020; 56.  5. Turtzo LC, McCullough LD. Sex differences in stroke. Cerebrovasc Dis. 2008; 26:462–74.  6. Roquer J, Campello AR, Gomis M. Sex differences in first-ever acute stroke. Stroke. 2003; 34:1581–5.  7. Guo X, Xiong Y, Huang X, Pan Z, Kang X, Chen C, et al. Sex-based differences in long-term outcomes after stroke: A meta-analysis. PLoS One. 2023; 18:e0283204.  8. Phan HT, Blizzard CL, Reeves MJ, Thrift AG, Cadilhac DA, Sturm J, et al. Factors contributing to sex differences in functional outcomes and participation after stroke. Neurology. 2018; 90:e1945-e1953.  9. Pantoja-Ruiz C, Akinyemi R, Lucumi-Cuesta DI, Youkee D, Emmett E, Soley-Bori M, et al. Socioeconomic Status and Stroke: A Review of the Latest Evidence on Inequalities and Their Drivers. Stroke. 2025; 56:794–805.  10. Marshall IJ, Wang Y, Crichton S, McKevitt C, Rudd AG, Wolfe CDA. The effects of socioeconomic status on stroke risk and outcomes. Lancet Neurol. 2015; 14:1206–18.  11. Shah RS, Cole JW. Smoking and stroke: the more you smoke the more you stroke. Expert Rev Cardiovasc Ther. 2010; 8:917–32.  12. Ohira T, Shahar E, Chambless LE, Rosamond WD, Mosley TH, Folsom AR. Risk factors for ischemic stroke subtypes: the Atherosclerosis Risk in Communities study. Stroke. 2006; 37:2493–8.  13. Autenrieth CS, Evenson KR, Yatsuya H, Shahar E, Baggett C, Rosamond WD. Association between physical activity and risk of stroke subtypes: the atherosclerosis risk in communities study. Neuroepidemiology. 2013; 40:109–16.  14. Harshfield EL, Georgakis MK, Malik R, Dichgans M, Markus HS. Modifiable Lifestyle Factors and Risk of Stroke: A Mendelian Randomization Analysis. Stroke. 2021; 52:931–6.  15. Zirnsak M, Meisinger C, Linseisen J, Ertl M, Zickler P, Naumann M, et al. Associations between pre-stroke physical activity and physical quality of life three months after stroke in patients with mild disability. PLoS One. 2022; 17:e0266318.  16. Matsuo R, Ago T, Kiyuna F, Sato N, Nakamura K, Kuroda J, et al. Smoking Status and Functional Outcomes After Acute Ischemic Stroke. Stroke. 2020; 51:846–52.  17. Zafar A. Diabetic patients are at a higher risk of lacunar infarction and dyslipidemia: results of a comparative pilot study from King Fahad Hospital of the University, Saudi Arabia. Neurosciences (Riyadh). 2017; 22:20–4.  18. Banerjee C, Chimowitz MI. Stroke Caused by Atherosclerosis of the Major Intracranial Arteries. Circ Res. 2017; 120:502–13.  19. Kato Y, Tsutsui K, Nakano S, Hayashi T, Suda S. Cardioembolic Stroke: Past Advancements, Current Challenges, and Future Directions. Int J Mol Sci. 2024; 25.  20. Amirzada M, Buczak-Stec E, König H-H, Hajek A. Multimorbidity patterns in the German general population aged 40 years and over. Arch Gerontol Geriatr. 2023; 114:105067.  21. Gallacher KI, Jani BD, Hanlon P, Nicholl BI, Mair FS. Multimorbidity in Stroke. Stroke. 2019; 50:1919–26.  22. Yousufuddin M, Young N. Aging and ischemic stroke. Aging (Albany NY). 2019; 11:2542–4.  23. Reddy HP, Jaganath A, Nagaraj N, Reddy VYJ. A study of age as a risk factor in ischemic stroke of elderly. Int J Res Med Sci. 2019; 7:1553.  24. Hanna M, Wabnitz A, Grewal P. Sex and stroke risk factors: A review of differences and impact. J Stroke Cerebrovasc Dis. 2024; 33:107624.  25. Peters SAE, Carcel C, Millett ERC, Woodward M. Sex differences in the association between major risk factors and the risk of stroke in the UK Biobank cohort study. Neurology. 2020; 95:e2715-e2726.  26. Patwardhan V, Gil GF, Arrieta A, Cagney J, DeGraw E, Herbert ME, et al. Differences across the lifespan between females and males in the top 20 causes of disease burden globally: a systematic analysis of the Global Burden of Disease Study 2021. Lancet Public Health. 2024; 9:e282-e294.  27. Phan HT, Reeves MJ, Blizzard CL, Thrift AG, Cadilhac DA, Sturm J, et al. Sex Differences in Severity of Stroke in the INSTRUCT Study: a Meta-Analysis of Individual Participant Data. J Am Heart Assoc. 2019; 8:e010235. | | | | |  |

| **Supplementary Table S3.** Sample characteristics of included and excluded patients | | | | | | |  |
| --- | --- | --- | --- | --- | --- | --- | --- |
| **Variable** | | **Included** | | **Excluded** | | **p** |  |
| Total, n | | 1,044 | | 680 | |  |  |
| Etiology,^1^ n (*col%)* | CE | 252 | (*24.1%*) | 172 | (*27.0%*) | 0.246^a^ |  |
|  | LAA | 226 | (*21.6%*) | 130 | (*20.4%*) |  |  |
|  | SVO | 184 | (*17.6%*) | 91 | (*14.3%*) |  |  |
|  | CC | 362 | (*34.7%*) | 220 | (*34.5%*) |  |  |
|  | OC | 20 | (*1.9%*) | 24 | (*3.8%*) |  |  |
|  | Missing Values | - |  | 43 |  |  |  |
| Age, Median (*IQR*^†^) | | 71.00 | (*60.0 - 78.0*) | 73.00 | (*60.0 - 81.0*) | 0.004^b^ |  |
| Alcohol,^2^ Median (*IQR*) | | 2.00 | (*1.0 - 4.0*) | 2.00 | (*0.0 - 4.0*) | < 0.001^b^ |  |
| BMI, Median (*IQR*) | | 26.5 | (*24.2 - 30.0*) | 26.1 | (*23.5 - 29.4*) | 0.020^b^ |  |
| Education,^3^ n (*col%*) | Low | 740 | (*74.6%*) | 365 | (*80.2%*) | 0.023^c^ |  |
|  | High | 252 | (*25.4%*) | 90 | (*19.8%*) |  |  |
| Employment status, n (*col%*) | Employed | 289 | (*28.2%*) | 114 | (*18.4%*) | < 0.001^c^ |  |
|  | Inactive | 736 | (*71.8%*) | 504 | (*81.6%*) |  |  |
| Multimorbidity,^4^ Median (*IQR*) | | 2.9 | (*1.9 - 5.0*) | 3.2 | (*1.6 - 5.6)* | 0.286^b^ |  |
| Physical Activity,^5^ n (*col%*) | Low | 516  278  250 | (*49.5%*)  (*26.6%*)  (*23.9%*) | 472  99  109 | (*69.4%*)  (*14.6%*)  (*16.0%*) | < 0.001^a^ |  |
|  | Moderate |  |  |  |  |  |  |
|  | High |  |  |  |  |  |  |
| Sex, n (*col%*) | Male | 619  425 | (*59.3%*)  (*40.7%*) | 369  310 | (*54.3%*)  (*45.7%*) | 0.048^c^ |  |
|  | Female |  |  |  |  |  |  |
| Smoking, n (*col%*) | Never | 463 | (*44.3%*) | 399 | (*58.7%*) | < 0.001^a^ |  |
|  | Former | 456 | (*43.7%*) | 193 | (*28.4%*) |  |  |
|  | Current | 125 | (*12.0%*) | 88 | (*12.9%*) |  |  |
| NIHSS admission,^6^ Median (*IQR*) | | 2.0 | (*0.0 - 4.0*) | 2.0 | (*0.0 - 5.0*) | 0.115^b^ |  |
| NIHSS discharge, Median (*IQR*) | | 0.0 | (*0.0 - 2.0*) | 1.0 | (*0.0 - 2.0*) | 0.009^b^ |  |
| **Note.**  ^1^ Etiology was classified according to the TOAST criteria: CE = cardioembolism; LAA = large-artery atherosclerosis;   SVO = small-vessel occlusion; CC = cryptogenic cause; OC = other cause.  ^2^ Alcohol consumption was measured using the Alcohol Use Disorders Identification Test.  ^3^ Education was assessed according to the ISCED-97 classification and dichotomized into low (ISCED levels 0–3) and high (ISCED levels 4–6).  ^4^ Multimorbidity was assessed using the summed beta coefficients of the Multimorbidity Weighted Index (MWI).  ^5^ Physical activity was assessed using the categorical score of the short-form International Physical Activity Questionnaire.  ^6^ Stroke severity was assessed by using the National Institutes of Health Stroke Scale (NIHSS).  ^a^ Pearson’s chi-squared test.  ^b^ Mann–Whitney U test.  ^c^ Pearson’s chi-squared test with Yates’ continuity correction.  ^†^ Interquartile range. | | | | | | |  |

| **Supplementary Table S4.** Fixed effects estimates from the linear mixed model | | | | |
| --- | --- | --- | --- | --- |
| **Variable** | | **β**^†^ **(***SE*^‡^**)** | **95% CI**^§^ | **p** |
| Intercept | | 95.33 (*2.05*) | [91.29, 99.37] | < 0.001 |
| Etiology^1^ | CE (*reference group*) |  |  |  |
|  | LAA | -0.46 (*1.83*) | [-4.05, 3.13] | 0.801 |
|  | SVO | -1.92 (*1.79*) | [-5.44, 1.61] | 0.286 |
|  | CC | 1.98 (*1.50*) | [-0.97, 4.92] | 0.150 |
|  | OC | 0.41 (*3.49*) | [-6.89, 7.70] | 0.909 |
| Age (*z-standardized*) | | 1.14 (*1.64*) | [-2.09, 4.37] | 0.488 |
| Age (*z-standardized*) squared | | -2.23 (*0.71*) | [-3.71, -0.75] | 0.005 |
| Age (*z-standardized*) cubed | | -2.53 (*0.70*) | [-3.93, -1.13] | < 0.001 |
| Age (*z-standardized*) quartic | | -0.55 (*0.18*) | [-0.94, -0.15] | 0.012 |
| Alcohol^2^ (*z-standardized*) | | 3.76 (*0.79*) | [2.20, 5.31] | < 0.001 |
| Alcohol (*z-standardized*) squared | | -1.41 (*0.38*) | [-2.17, -0.65] | < 0.001 |
| BMI (*z-standardized*) | | -1.51 (*0.62*) | [-2.75, -0.27] | 0.018 |
| Education^3^ | High (*reference group*) |  |  |  |
|  | Low | -3.15 (*1.27*) | [-5.65, -0.64] | 0.014 |
| Employment status | Employed (*reference group*) |  |  |  |
|  | Inactive | -4.04 (*1.83*) | [-7.65, -0.42] | 0.029 |
| Multimorbidity^4^ (*z-standardized*) | | -4.39 (*0.88*) | [-6.13, -2.66] | < 0.001 |
| Multimorbidity (*z-standardized*) squared | | 0.71 (*0.25*) | [0.19, 1.23] | 0.010 |
| Physical activity^5^ | Moderate (*reference group*) |  |  |  |
|  | Low | -4.08 (*1.28*) | [-6.60, -1.56] | 0.002 |
|  | High | 0.42 (*1.35*) | [-2.24, 3.08] | 0.758 |
| Sex | Male (*reference group*) |  |  |  |
|  | Female | -1.14 (*1.31*) | [-3.72, 1.44] | 0.385 |
| Smoking^6^ | Never (*reference group*) |  |  |  |
|  | Former | -2.83 (*1.28*) | [-5.35, -0.31] | 0.028 |
|  | Current | 0.08 (*1.75*) | [-3.37, 3.53] | 0.965 |
| **Note.**  ^1^ Etiology was classified according to the TOAST criteria:   CE = cardioembolism; LAA = large-artery atherosclerosis; SVO = small-vessel occlusion; CC = cryptogenic cause; OC = other causes..  ^2^ Alcohol consumption was measured using the Alcohol Use Disorders Identification Test.  ^3^ Education was assessed according to the ISCED-97 classification and dichotomized into low (ISCED levels 0–3) and high (ISCED levels 4–6).  ^4^ Multimorbidity was assessed using the summed beta coefficients of the Multimorbidity Weighted Index (MWI).  ^5^ Physical activity was assessed using the categorical score of the short-form International Physical Activity Questionnaire.  ^†^ Beta Regression Coefficient.  ^‡^ Standard Error (cluster-robust CR2 type).  ^§^ Confidence Interval. | | | | |

| \| **Supplementary Figure S1**. Directed acyclic graph illustrating assumed causal relationships \| \| --- \| \| **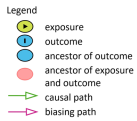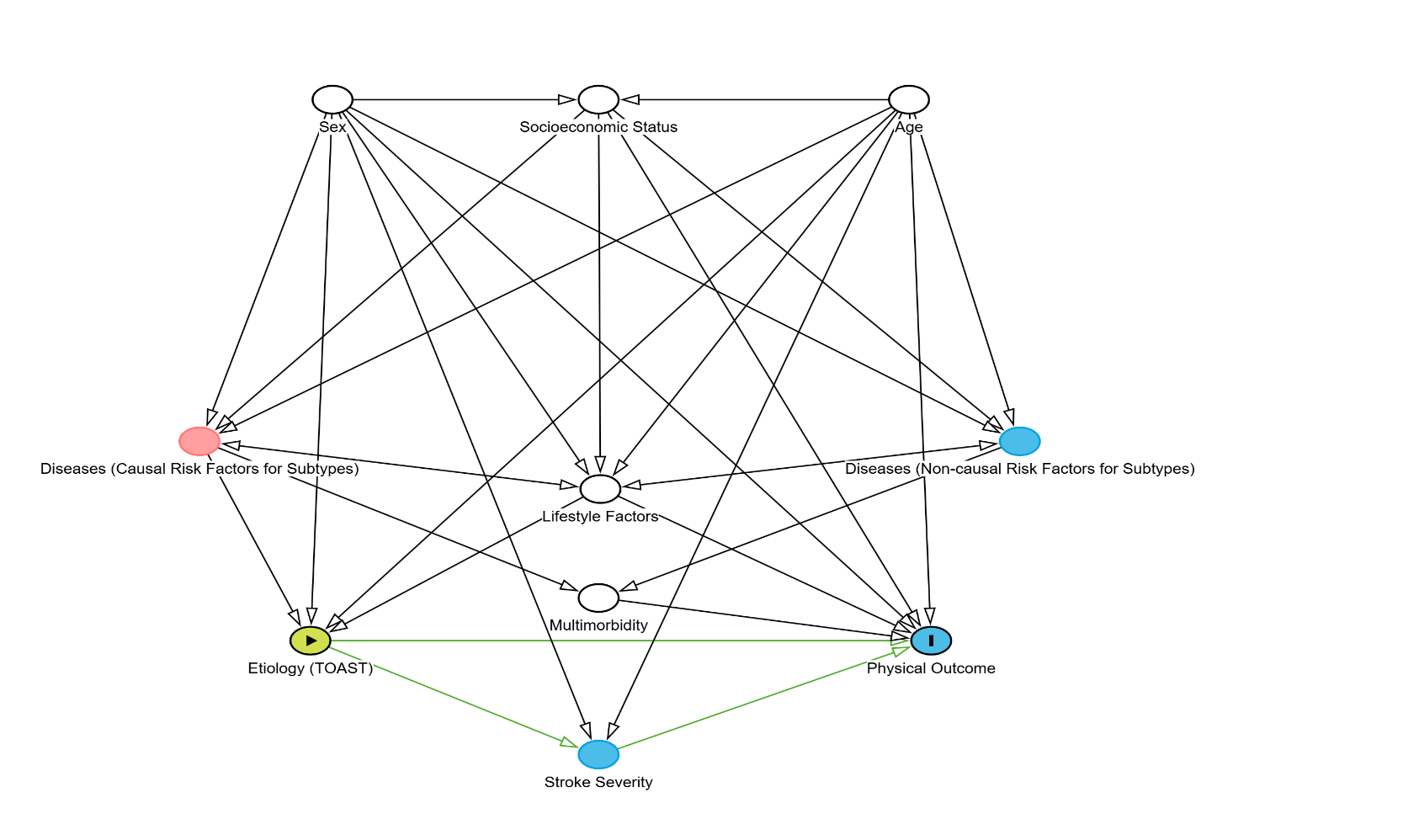** \| |
| --- | --- | --- |

| **Supplementary Figure S2.** Histograms of SIS domain scores |
| --- |
| 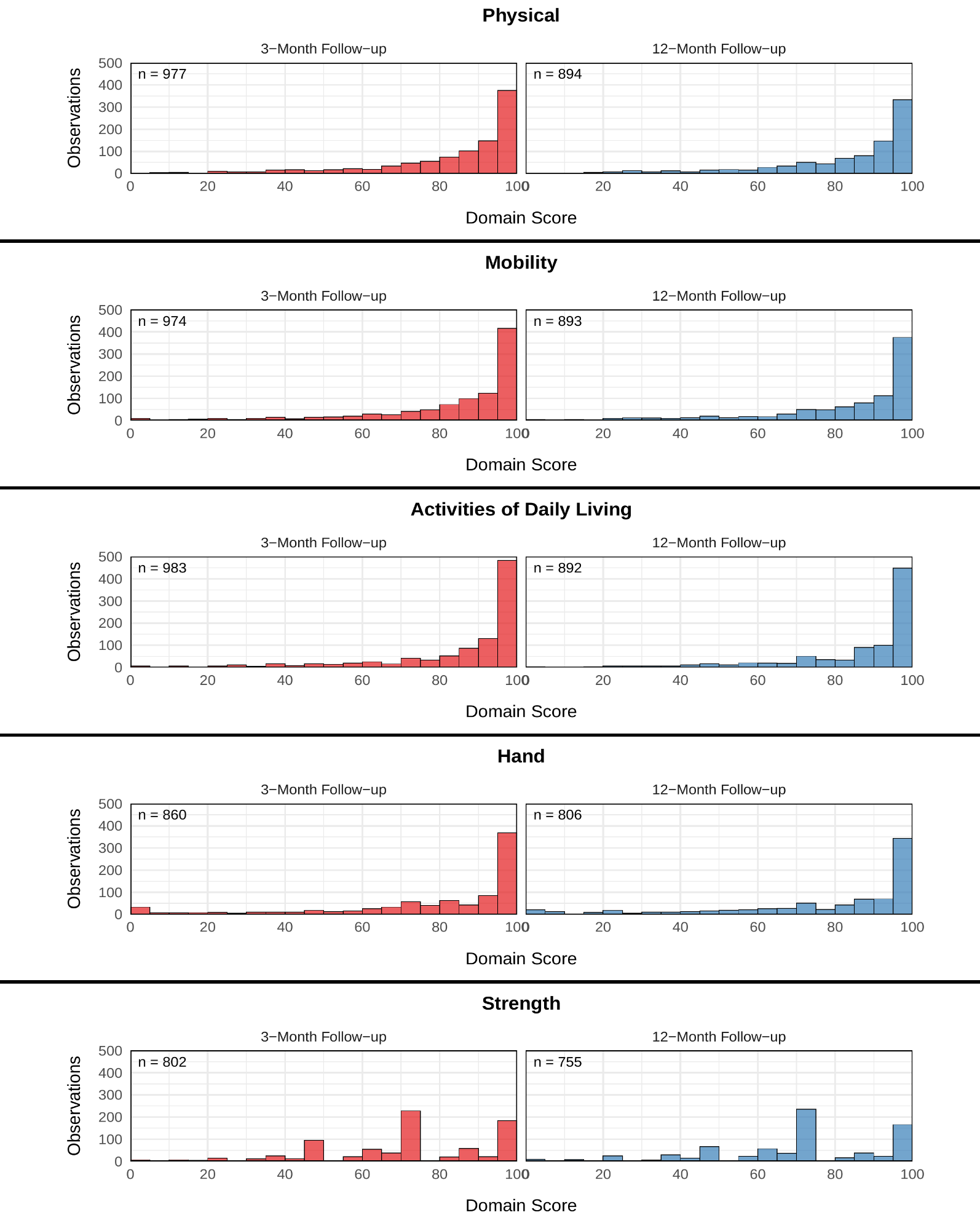 |
